# Sorption of Neuropsychopharmaca in Microfluidic Materials for *In Vitro* Studies

**DOI:** 10.1021/acsami.1c07639

**Published:** 2021-09-16

**Authors:** Thomas E. Winkler, Anna Herland

**Affiliations:** † Division of Micro- and Nanosystems, KTH Royal Institute of Technology, 10044 Stockholm, Sweden; ‡ AIMES, Center for Integrated Medical and Engineering Science, Department of Neuroscience, Department of Neuroscience, Karolinska Institute, Solna 17165, Sweden

**Keywords:** organs-on-chips, non-specific binding, microfluidics, materials, neuropsychopharmaca

## Abstract

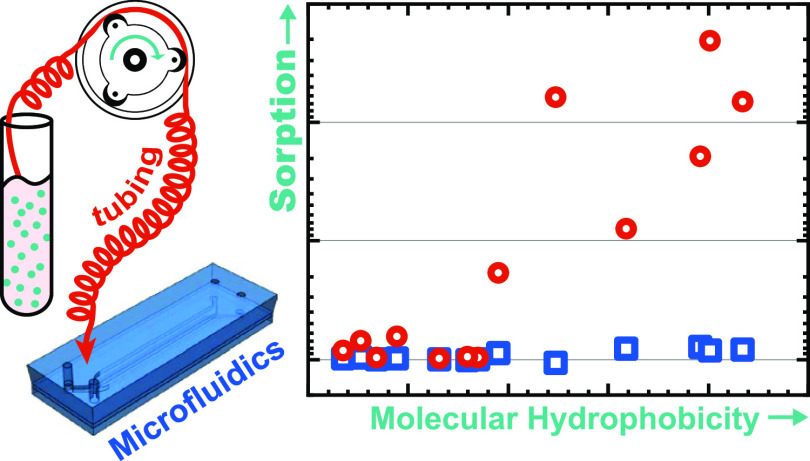

Sorption (*i.e.*, adsorption and absorption) of small-molecule compounds to polydimethylsiloxane (PDMS) is a widely acknowledged phenomenon. However, studies to date have largely been conducted under atypical conditions for microfluidic applications (lack of perfusion, lack of biological fluids, etc.), especially considering biological studies such as organs-on-chips where small-molecule sorption poses the largest concern. Here, we present an in-depth study of small-molecule sorption under relevant conditions for microphysiological systems, focusing on a standard geometry for biological barrier studies that find application in pharmacokinetics. We specifically assess the sorption of a broad compound panel including 15 neuropsychopharmaca at *in vivo* concentration levels. We consider devices constructed from PDMS as well as two material alternatives (off-stoichiometry thiol–ene–epoxy, or tape/polycarbonate laminates). Moreover, we study the much neglected impact of peristaltic pump tubing, an essential component of the recirculating systems required to achieve *in vivo*-like perfusion shear stresses. We find that the choice of the device material does not have a significant impact on the sorption behavior in our barrier-on-chip-type system. Our PDMS observations in particular suggest that excessive compound sorption observed in prior studies is not sufficiently described by compound hydrophobicity or other suggested predictors. Critically, we show that sorption by peristaltic tubing, including the commonly utilized PharMed BPT, dominates over device sorption even on an area-normalized basis, let alone at the typically much larger tubing surface areas. Our findings highlight the importance of validating compound dosages in organ-on-chip studies, as well as the need for considering tubing materials with equal or higher care than device materials.

## 1 Introduction

Over the past decades, microfluidic devices and systems have moved from academia to the translational realm.^1^ At the same time, particularly academic applications have introduced more and more biological elements into microfluidic systems, starting with blood for point-of-care testing to complex cell ensembles for recapitulating human organ functions *in vitro*.^2−4^ Such applications impose ever more rigorous requirements on the materials used in device construction.^5,6^ One major criterion is whether the material alters the behavior or function of the biological element(s) that is to be assessed, that is, its “biocompatibility”. Thermoplastics (preferred for commercial production) and polydimethylsiloxane (PDMS; preferred in academic labs) generally perform well on this metric, though a case-by-case assessment is needed. Another important consideration is whether the material interferes with the chemical compounds to be tested, regardless if talking about a point-of-care biosensor or about drug testing using organs-on-chips.

Studies around the issue of PDMS compound sorption—that is, non-specific absorption and adsorption, particularly of small molecules (200–500 Da)—started appearing as early as 2001.^7^ Attention to the phenomenon increased as the microfluidics field continued to expand, perhaps most notably after a widely cited 2006 study considering Nile red and quinine (two small-molecule fluorophores).^8^ It has become accepted that PDMS is capable of significant sorption of small molecules—especially highly hydrophobic ones (log *P* > 2)^9−14^—not only onto its surface but also into its polymer matrix (acting as a “solid solvent”).^15^ Depending on the molecule in question, some fraction is subsequently released back into solution over the course of seconds (mainly for surface adsorption) to hours and days (mainly for bulk absorption).^9,11,16,17^ PDMS thus acts as a kind of capacitor for compound concentrations. With steady-state experiments, mathematical and computational models have shown some promise toward accounting for these effects in pharmacokinetic and pharmacodynamic studies.^18,19^ With more complex dynamic compound-dosing studies, however, this becomes difficult if not impossible. The aggregate research on compound sorption has driven studies into limiting such sorption by PDMS surface treatments^7,9,10,20−24^ and into the use of alternate microfluidic materials^12−14,25−28^—the latter being further motivated by PDMS’ other limitations, for example, regarding industrial scale-up.^5,6^

In surveying the aggregate body of research, we can, however, identify three shortcomings in prior studies regarding compound sorption with PDMS and other microfluidic materials, which our present paper will seek to address as follows: (1) lack of biologically relevant fluids, (2) generalization from few compounds, and (3) disregard for the overall fluidic circuit.

First, most studies employ simple buffer solutions; only a few utilize cell culture media—but in either case without proteins/serum. The one exception we have been able to identify found that PDMS sorption was unaffected by the absence/presence of serum but considered only a single molecule (estradiol).^29^ Protein-/serum-free conditions are unlikely to apply to biological sample analysis and are generally unsuitable for *in vitro* cell culture. Abundant proteins such as serum albumin, however, are likely to adsorb onto surfaces^30^ and (i) alter their contact angle, for example, in the case of PDMS, rendering it more hydrophilic,^31^ thereby potentially lowering the hydrophobic interactions with free hydrophobic compounds and (ii) physically restrict the access of small molecules to the surface, potentially decreasing any sorption-related interactions.

Second, much of the prior research focuses on mitigation (*via* materials, coatings, models, ...). These studies often employ one to three “model” compounds (typically fluorophores such as FITC or rhodamine B) and then generalize to other molecules based on the assumption that sorption behavior is largely similar between molecules of similar size and hydrophobicity. As we will illustrate, however, even the smaller body of quantitative multi-compound characterization studies^9−14^—to which our study contributes—does not provide a solid basis for such assumptions.

Third, microfluidic sorption is considered with a practically exclusive focus on the device itself.^32^ In many scenarios, however, the device represents only a small fraction of the total wetted surface area; liquid reservoirs and especially fluidic tubing (where external pumps are needed) will dominate this by a factor of >10. The assumption of these materials’ irrelevance to study outcomes is perhaps best exemplified by their lack of specification in many Materials and Methods sections. The fact that sorption goes beyond microfluidic materials is implicitly recognized in applied pharmacological studies, where non-specific sorption of the entire system is often measured^33−35^—but the results generally do not translate to other systems. Explicit focus on, and recognition of, the importance of fluidic tubing in the context of microfluidic studies appears limited to a largely overlooked 2009 study, which considered one common type of tubing (PharMed BPT) with three compounds and reported significant compound loss in the tubing (along with some mitigation strategies).^36^

Herein, we present the characterization of compound sorption under conditions closely mimicking biological microfluidics, particularly those encountered during *in vitro* drug testing in a barrier-on-chip system. Within this context, our study seeks to specifically address the three shortcomings discussed above. We consider a broad panel of 18 small-molecule compounds—mainly clinically relevant neuropsychopharmaca—with a range of physicochemical properties. With these, we characterize material sorption over a typical 24 h exposure in a complete (protein-supplemented) cell culture medium (CCM). We prepare microfluidic devices matching a standard design (vertically stacked co-linear channels)^37^ from (1) PDMS as well as two suitable alternatives: (2) off-stoichiometry thiol–ene–epoxy (OSTE+), a polymer system with highly desirable bonding/assembly properties and which features a largely inert and hydrophilic surface after processing;^25^ and (3) thermoplastics in combination with a double-coated pressure-sensitive adhesive tape (PSA) to facilitate microfluidic device assembly/bonding.^38−40^ Moreover, we compare the material sorption of three widely available peristaltic pump tubings suitable for biomicrofluidic applications:^41^ polypropylene-based (PharMed BPT), silicone-based (Tygon SI), and polyolefin-based (Tygon MHLL).

## 2 Device and Study Design

**Figure 1:**
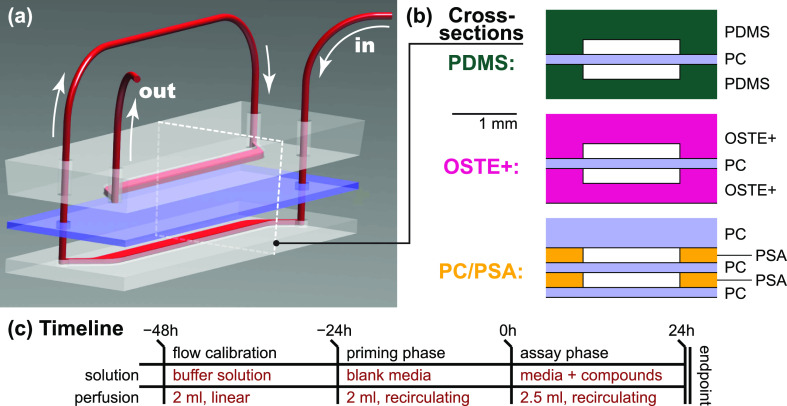
Barrier-on-chip-type microfluidic devices. (a) Schematic 3D rendering, showing the media perfusion (red) through the microfluidics from the in(let) to the out(let). The internal wetted microfluidic area is 1 cm^2^. White dotted line indicates the cross-sectional plane. (b) Cross sections for the three material options we consider in our study. (c) Experimental timeline showing the solutions, volumes, and perfusion types employed. Abbreviations: PDMS—polydimethylsiloxane; OSTE+—off-stoichiometry thiol–ene–epoxy; PC—polycarbonate; PSA—pressure-sensitive adhesive.

Our microfluidics feature dimensions typical for barrier-on-chip research with a cross section of 1.5 × 0.2 mm^2^ (*w* × *h*).^42,43^ We followed one of the most established barrier-on-chip designs by placing two such channels on top of each other.^4,18,39,44^ Typically, they would be separated by a porous membrane serving as a cell culture support to facilitate biological barrier formation. Such membranes can be fabricated from structurally matched materials such as PDMS or OSTE+,^45,46^ or (as is most common and widely accessible) obtained commercially from polyethylene terephthalate (PET) or polycarbonate (PC). To simplify our experimental design and to focus on sorption by major structural materials rather than the membrane itself, we, however, replaced it with a non-porous PC film. PC can generally be regarded as low-sorbing, similar to polypropylene and cyclic olefin copolymer (COC).^12,47^ The resulting devices and flow paths, which enabled us to utilize just one pump channel per device, are illustrated in Figure 1a,b. Each pump channel perfused a “material of interest” surface area (*i.e.*, disregarding the PC divider) of 1 cm^2^, which matches the typical barrier-on-chip designs (including their permeable membranes). For perfused systems like ours, however, this constitutes only a small part of the total wetted surface area, which additionally consisted of 7.1 cm^2^ tubing, 0.8 cm^2^ stainless-steel interconnects, and ∼7 cm^2^ fluid reservoirs (at a typical fill volume).

The epithelial or endothelial cells typically employed in barrier-on-chip devices can greatly benefit from physiologically relevant shear (τ = 1–10 dyne cm^–2^).^48−50^ In human capillaries, flow rates *Q* < 1 μL h^–1^ are sufficient.^51^ To achieve similar forces in microfluidic geometries, we consider the following equation (suitable for *w* > *h*)
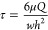
1

With geometries in the hundreds of microns, this means that milliliters per hour are required (even when adjusting the viscosity μ to *in vivo*-like values of 3–4 mPa s).^52^ With a multiplexed, multi-day experiment, this adds up to an unsustainable volume of media (and associated amounts of drugs or similar test compounds), thus necessitating media recirculation. These requirements (recirculation and multiplexing) make peristaltic pumps the obvious (and in most cases, only) answer.^53^ Only a limited variety of tubing materials is available, however;^41^ the selection is even more limited when requiring biocompatibility as per USP Class VI or ISO 10993 (reasonable minimum standards for *in vitro* applications). This leaves us with three classes of materials (we use market-dominant Tygon plastics; alternative manufacturers’ products will still fall into the same categories): polypropylene-based (PharMed BPT), silicone-based (Tygon SI), and polyolefin-based (Tygon MHLL). PVC-based tubing (*e.g.*, Tygon LFL) may be USP-certified but is not recommended for usage with blood or tissues due to plasticizer leaching concerns. For device studies, we employed PharMed BPT due to its widespread use; for tubing evaluation, we constructed flow circuits of equal length from all tubing materials, omitting only the microfluidic devices.

Our experimental timeline, shown in Figure 1c, closely mirrors that of on-chip *in vitro* studies.^4,39^ Before use, we plasma-treated devices and tubing to facilitate wetting and disinfected them by flushing with 70% ethanol. The initial 24 h period, with phosphate buffered saline (PBS) perfusion, then served to calibrate flow rates and check for leakage. We subsequently switched to recirculating perfusion of the cell culture medium (with 10% serum replacement unless noted) at τ = 1.1 dyne cm^–2^ to equilibrate the fluidics with the test fluid. The third day constitutes the actual assay phase, where we introduced the test compounds of interest into the system. After continuous perfusion over another 24 h, samples were collected and analyzed (endpoint).

As sorption test compounds, we selected 3 fluorophores and 15 neuropsychopharmaca (Table 1). Brain-targeting drugs with their generally high hydrophobicity (to ease passage across the blood–brain barrier)^54^ present a particularly difficult scenario. The specific group of compounds was selected based on (i) their known clinical need for therapeutic drug monitoring (TDM),^55^ as this can indicate the relevance of *in vitro* time-course studies as well as the desirability of labs-on-chips for point-of-care analysis; (ii) their spread in hydrophobicity to encompass a range of properties;^56^ and (iii) the non-restricted availability of the compounds themselves, as well as of standardized clinical TDM analysis. Overall, their properties are representative of brain-targeting drugs in general (mean mass, *M* ∼ 310 Da; hydrogen bond donor count H-Bd < 3), with a bias toward higher hydrophobicity (literature median log *P*: 2.5; ours: 3.7) to increase the likelihood of observing material sorption.^57^ Combined with concentrations *C* at 5/6th of the upper therapeutic range limit in plasma,^55^ our compound panel represents realistic conditions for pharmacokinetic studies in a barrier-on-chip system.

**Table 1:** Compounds Selected for Study, as Well as Relevant Physical and Chemical Properties^a^

class^b^	compound	TDM^b^	*C*^b^ [g L^–1^]	*M*^c^ [Da]	Log *P*^c^	TPSA^c^ [nm^2^]	H-Bd^c^
anti-convulsant	lacosamide (La)	+	8.3 × 10^–3^	250	–0.02	0.674	2
	licarbazepine (Li)	++	2.9 × 10^–2^	254	1.73	0.666	2
	rufinamide (Ru)	++	2.5 × 10^–2^	238	1.27	0.738	1
	zonisamide (Zo)	++	3.3 × 10^–2^	212	0.11	0.862	1
anti-depressant	amitriptyline (Am)	+++	1.7 × 10^–4^	277	4.81	0.032	0
	citalopram (Ci)	+++	9.2 × 10^–5^	324	3.76	0.363	0
	clomipramine (Cl)	+++	3.8 × 10^–4^	315	4.88	0.065	0
	fluoxetine (Fl)	+	4.2 × 10^–4^	309	4.17	0.213	1
	nortriptyline (No)	+++	1.4 × 10^–4^	263	4.43	0.120	1
	sertraline (Se)	++	1.3 × 10^–4^	306	5.15	0.120	1
	vortioxetine (Vo)	++	3.3 × 10^–5^	298	4.76	0.153	1
anti-psychotic	haloperidol (Ha)	+++	8.3 × 10^–6^	376	3.66	0.405	1
	paliperidone (Pa)	++	5.0 × 10^–5^	426	1.76	0.822	1
	risperidone (Ri)	++	5.0 × 10^–5^	410	2.63	0.619	0
anti-addictive	methadone (Me)	++	5.0 × 10^–4^	309	5.01	0.203	0
fluorophore	fluorescein (F*)		2.8 × 10^–4^	330	3.01	0.895	0
	Nile blue (N*)		2.7 × 10^–4^	318	3.85	0.504	1
	TAMRA (T*)		3.6 × 10^–4^	430	–0.29	0.929	1

a TDM, therapeutic drug monitoring; *C*, concentration in our study; *M*, molar mass; *P*, octanol–water partition coefficient; TPSA, topological polar surface area; H-Bd, H-bond donor count; and TAMRA, carboxytetramethylrhodamine.

b Classifications and TDM ratings [from potentially useful (o) to strongly recommended (+++)] are adapted from Hiemke *et al.*, whose upper therapeutic range limits (in plasma) we used to select concentrations (at 5/6th).^55^

c Physical properties are derived from ChemAxon Chemicalize.^56^

## 3 Results and Discussion

### 3.1 Microfluidic Devices

**Figure 2:**
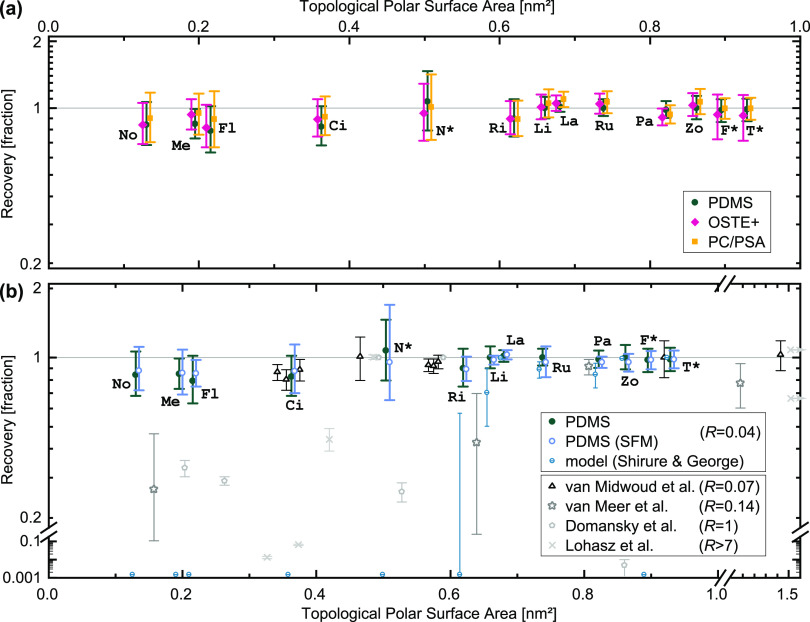
Sorption in microfluidic devices. Compound recovery from microfluidic devices after 24 h of continuous recirculating perfusion, expressed as a fraction of tubing-only control concentrations (*i.e.*, 1 equating zero loss inside devices). Compounds are sorted along the *x*-axis by their hydrophobicity in terms of topological polar surface area (offset for better visualization where needed; for abbreviations, see Table 1). Unless otherwise noted, we use a complete cell culture medium (includes 10% serum). (a) Comparison of device materials as listed in the legend and illustrated in Figure 1. (b) Comparison of PDMS results for medium with and without proteins/serum (SFM: supplement-free medium; otherwise 10% serum replacement). Also in blue, but with smaller symbols, we indicate predictions for our experiment generated using a model.^58^ Data from four other experimental studies (see Table 2 and text) are included for further comparison with distinct symbols in shades of gray.^10,12−14^*R* denotes the ratio of microfluidic surface area to total liquid volume. Experimental data are plotted as means (*n* = 4 per condition), with error bars representing the 95% confidence interval. The model error bars represent a range of assumptions for molecular diffusivity inside PDMS (see PDMS Sorption Model, Section 5.7). For literature values, error bars represent SD (*n* = 3)^12,14^ or range (*n* = 2),^10,13^ and arrows on the right border indicate protein-size compounds.

First, we consider compound sorption for our microfluidic devices. To extract only the device contribution, we compare all results to corresponding tubing-only experiments (*i.e.*, identical fluidic circuits omitting the devices). This corrects for device material-independent losses such as thermal degradation (Figure S1) or sorption by other parts of the setup (discussed later in Section 3.2). We plot the resulting compound recovery fraction, that is, how much of the molecules of interest remained in solution, in Figure 2a for various microfluidic materials. Along the *x*-axis, we sort compounds by their topological polar surface area (TPSA), a measure of hydrophilicity and size (lower TPSA = smaller and more hydrophobic). The choice is based on van Meer *et al.*’s finding wherein PDMS sorption correlated better with TPSA than with the octanol–water partition coefficient log *P*, the more commonly used hydrophobicity metric.^10^ It is also a good conceptual match for the model proposed by Shirure and George, where PDMS sorption is assumed to be governed by both size and hydrophobicity.^58^ An added practical advantage of TPSA is that calculations are less divergent between prediction algorithms (compared to log *P*).

Of the 13 compounds detected (*cf.*Section 3.2), we find that—independent of the device material—all of their 95% confidence intervals envelop a recovery efficiency of 1.0 (*i.e.*, zero compound loss/sorption). The per-compound confidence intervals also strongly overlap between material conditions. We note, however, a decrease in means toward the left side of the plot, suggesting potential trends when considering all compounds. Linear regression analysis confirms that sorption noticeably increases with increasing hydrophobicity for PDMS as well as OSTE+ (but not PC/PSA; the relevant data are included as part of the overall summary in Section 3.2).

Putting the PC/PSA result in context is difficult. PC, which accounts for over two-thirds of the active fluidic surface area in these devices, is known to be a comparatively low-sorbing plastic on par with polypropylene or COC (all superior to *e.g.*, PDMS).^12,47^ The inherently sticky PSA, however, could *ab initio* be a cause for concern. The one extant study we were able to locate on PSA sorption only provides qualitative analysis for a single fluorescent dye.^59^ They observed limited sorption of dye, especially in the presence of proteins, under ambient conditions. This is very broadly in agreement with our own findings, apparently the first quantitative ones involving PSA. With our PC/PSA exhibiting the lowest overall sorption among fluidic materials, the impact of PSA is at the very least not high enough to outweigh the benefits of a low-sorbing thermoplastic constituting the majority of the microfluidic surface area. Even if the confidence intervals overlap the other material conditions, the trend analysis suggests that it could be considered an optimal choice from a sorption standpoint. We note that PSA lamination is also one of the simplest ways to realize glass-based devices, as demonstrated by Kratz *et al.*(38) Compared to PC, glass would further lower molecular sorption,^16,47,60^ albeit at the expense of practical advantages (robustness and chip-to-world interfacing).

OSTE+ sorption has previously been studied qualitatively and quantitatively but only with rhodamine B in water.^61−63^ These findings generally showed that OSTE+, unlike PDMS, does not exhibit bulk absorption (as expected for a tightly crosslinked matrix), but that it does feature noticeable surface adsorption (a finding that we replicate here; Figure S2). This still presents a significant potential advantage, since surface sites can saturate and also be modified with appropriate surface chemistry. PDMS devices, conversely, can continue to absorb compounds into loosely crosslinked internal volumes many times that of the microfluidic channels they contain. Our own results for OSTE+ cannot establish whether it has any absolute advantages over PDMS regarding compound sorption. However, based on the hydrophobicity trend analysis, it performs at least similarly to PDMS (which itself performs well), or better.

PDMS allows for the most expansive discussion, due to a larger existing body of research. In our study, the lower ends of the confidence intervals all remain above 0.6. Our findings do indicate that hydrophobicity impacts the sorption behavior of PDMS, but only with a small effect size, and without any outliers. This is significantly lower sorption than the prediction we generated using a model by Shirure and George (Figure 2b), which mainly considers device geometry, molecular size, and log *P*.^58^ It is also a significant contrast to prior experimental studies (*cf.*Table 2 and Figure 2b), which generally report sorption in excess of 0.5 log_10_ (∼30%) for at least half of their tested compounds below a proposed TPSA threshold for sorption of 0.9 nm^2^.^9−11,13^ To explain why some (but not all) compounds below the cutoff exhibited high sorption, Auner *et al.* consider 15 chemical and physical descriptors.^11^ They suggest that, below the TPSA cutoff, the number of H-bond donors H-Bd—potentially related to molecular diffusivity within PDMS—becomes the determining factor (high sorption if 0; also more likely to exhibit high sorption at 1 and potentially at 2). However, as per Table 1, ten of our measured compounds here fall below the TPSA cutoff and satisfy H-Bd < 2 (Me, Ci, and Ri at 0). This suggests that the H-bd criterion may have arisen from compound selection bias. It is worth noting that Auner *et al.*’s study (the only one to exceed ours in compound number) focused on toxins rather than (as in our study) pharmaceuticals, also covering a range of more hydrophilic molecules, as well as compounds with H-Bd > 2.^11^

**Table 2:** Overview of Studies on PDMS Compound Sorption^a^

study (main class of compounds studied)	compounds: total^b^	TPSA < 0.9 nm^2^, H-Bd < 2^b^	recovery < 0.7^b^	solution	non-PDMS controls^c^	plasma treatment	study type^d^	perfusion^e^	duration *t* [h]^f^	liquid volume [μL]	surface area [cm^2^]	ratio *R* [mm^–1^]
present work (neural drugs)	13^g^	10	**0**	CCM or SFM	√	√	μF	↶	24	2500	1	0.04
present work (model Shirure & George)^58^	13^g^	10	**7**						24	2500	1	0.04
Auner *et al.*(11) (cytotoxins)	19	9	**3**	PBS			I		24+	2500	0.8	0.03
van Midwoud *et al.*(12) (liver metabolites)	9	4	**0**	SFM	√	√	μF	→	2	600	0.4	0.07
van Meer *et al.*(10) (cardiac drugs)	4	3	**2**	PBS	√		I		3	250	0.3	0.14
Domansky *et al.*(14) (varied)	6	4	**4**	PBS	√	?^h^	I		72	30	0.3	1.0
Lohasz *et al.*(13) (varied)	6	3	**3**	SFM	√	?^h^	μF	↶	10+	150	>10	>7
Wang *et al.*(9) (varied)	5	1	**2**	PBS		√	μF		4.5	15	1.7	11
Auner *et al.*(11) (cytotoxins)	13	5	**3**	PBS			μF		6+	<13	<0.9	12

a The table summarizes some of the relevant parameters of the studies. We excluded studies with quantitative results for less than three compounds. Values are estimated from available data where not explicitly stated in the relevant papers. *R*, surface area-to-liquid volume ratio.

b Number of compounds in total; meeting Auner *et al.*’s proposed criteria of TPSA < 0.9 nm^2^ and H-Bd < 2;^11,56^ and with PDMS sorption > 30%.

c Non-PDMS controls are required to account for, for example, thermal degradation losses, and for inclusion in Figure 2b.

d Microfluidic (μF) or disk immersion (I)-type experiments.

e Recirculating ↶ or linear →.

f For time-resolved studies which continued measuring beyond where steady-state conditions were achieved, we denote the typical duration to the steady state instead (+).

g Though our study includes 18 compounds in total, sorption quantification in devices is only possible for 13 due to the compounding losses discussed in Section 3.2.

h Question marks (?) denote works where plasma treatment was studied, but where it remains unclear whether it was applied for the sorption measurements.

For additional context, we first consider the various time/volume/surface parameters from prior research. We would expect a shorter exposure duration *t* and a lower PDMS surface-to-liquid volume ratio *R* to correlate with lower sorption. These assumptions are formalized in the model by Shirure and George, wherein sorption is proportional to *R* and √*t* (*cf.* PDMS Sorption Model Section 5.7).^58^ We note that the flow rate *Q* enters the equation only in terms of time *t* over total liquid volume (contained within *R*). Linear perfusion of a certain volume can be considered equivalent to multiple recirculations of the same volume with higher speed; the molecules of interest will ultimately reach the same global equilibrium between solution and microfluidics (though local phenomena may differ), as the microfluidic residence time of a given liquid volume is overall the same. *R* clearly divides the studies in Table 2 into two groups (Domansky *et al.*’s work,^14^ notwithstanding). Those with *R* ∼ 10 mm^–1^ would all be expected to show higher sorption based on this fact alone, and comparison to those with 100-fold lower *R* ∼ 0.1 mm^–1^ should almost certainly be avoided. Indeed, the fraction of high-sorption compounds is higher in the latter group (Table 2). Even within the low-*R* group, to which our study belongs, however, the reported differences in sorption behavior are sizable. We will attempt to address some of the potential confounding factors in the following paragraphs: sample solution type, plasma treatment, inclusion of non-PDMS controls, and finally a closer look at time and *R*.

One difference in our study compared to prior research is the, for *in vitro* modeling realistic, use of CCM rather than SFM, or buffer solutions (PBS). Since we include both CCM and SFM conditions (Figure 2b), with SFM exhibiting practically identical trends, we can, however, rule out the protein content as a dominant factor in PDMS compound sorption. This refutes one of our initial hypotheses (that high PDMS compound sorption in prior studies is due to the use of SFM/PBS instead of CCM) and provides supporting evidence for this previously estradiol-only finding.^29^ In terms of sample solutions, Table 2 also shows that studies finding high sorption in the low-*R* group rely on PBS rather than even on SFM. However, the SFM example in the high-*R* group shows some of the highest sorption overall. Together with the generally hydrophilic nature of constituents that differentiate SFM from PBS (therefore unlikely to alter the hydrophobic sorption behavior),^64^ we conclude that the use of SFM *versus* PBS is unlikely to explain the range of sorption behavior observed.

Another notable difference between studies in Table 2 is the use of air/oxygen plasma pre-treatment used to clean PDMS devices and temporarily render them more hydrophilic for simpler initial perfusion. Many extant studies (also beyond those in Table 2) consider untreated PDMS even in microfluidic test formats,^8,11,13,16,29^ whereas we include this as a step typically performed prior to realistic use. Auner *et al.* speculated that plasma treatment could in fact play a role in PDMS compound sorption (a more hydrophilic surface could decrease the hydrophobic molecule sorption).^11^ However, Wang *et al.* employed plasma treatment in an otherwise nearly identical study design to Auner *et al.*’s microfluidic experiments—and still observed sorption that largely aligned with that of Auner *et al.*(9,11) This is a strong indication that plasma treatment does not have an impact on the PDMS sorption behavior. Another argument for the lack of effect lies in the relevant dynamics. After plasma treatment, the PDMS surface angle reverts back to 80% of its initial value over 48 h—^65,66^ that is, for our present study, the delay between plasma exposure and pharmaceutical exposure. Plasma treatment, like any other vacuum-based process, also extracts dissolved atmospheric gases from inside the PDMS matrix; these, however, re-equilibrate on an even shorter timescale of minutes to hours.^67,68^ Last but not least, in our later results with silicone-based tubing (Section 3.2)—a material very similar to PDMS—we do not observe any changes in compound sorption with the inclusion or omission of plasma treatment. Taken together, this points to negligible effects from plasma exposure on compound sorption.

We find a more important caveat in that prior studies (except Domansky *et al.*)^14^ report compound loss relative to starting concentrations.^9−13^ Potential losses from inherent compound degradation (due to time, temperature, *p*O_2_, ...; *cf.*Figure S1) are thus conflated with PDMS sorption. In two studies (Lohasz *et al.* and van Midwoud *et al.*), control data with thermoplastic materials can establish that such losses were negligible.^12,13^ No such data were provided by Wang *et al.* or Auner *et al.* (closest to our study in *R*).^9,11^ In van Meer *et al.*’s experiments, a comparison to those control values (rather than initial) decreases the reported PDMS sorption figures by up to half.^10^ The data displayed in Figure 2b include the appropriate corrections.

Considering all relevant parameters, we can say that our study is most similar to the one by van Midwoud *et al.*, and indeed theirs is the only other multi-compound study finding PDMS sorption in a range (0–20%; Figure 2b) similar to our own data.^12^ Although our study lags behind van Midwoud *et al.*’s in terms of *R*, we may still have expected a 2-fold larger effect size than their study based on our longer liquid residence times (using the *R*√*t* proportionality). Van Meer *et al.*’s study also points to a larger expected effect size (even accounting for inherent losses mentioned above), since their larger *R* should be compensated for by our longer duration.^10^ The nature of the experiment (disk immersion) is naturally different, but diffusion (∼1 cm/24 h) limits the PDMS-exposed molecules per unit time similarly as perfusion does in microchannel experiments like ours. Lastly, the model by Shirure and George^58^ similarly predicts a larger effect size for our study design.

Our PDMS data, and the above discussion, point to PDMS sorption being strongly dependent on *R* as well as on the specific compounds chosen. PDMS clearly exhibits compound sorption behavior based on hydrophobicity in terms of TPSA. However, the effect size—for *R* in line with a typical *in vitro* study—is smaller than generally assumed. Exceedingly high-sorption compounds (>30%) appear to be outliers, rather than the norm, even when only considering hydrophobic ones (as also evidenced by the lack of clear TPSA dependence in the Domansky *et al.* and Lohasz *et al.* data in Figure 2b). None of the criteria and molecular properties suggested to date seem sufficient to predict these with certainty. This also presents a limitation for proposed models, which are extrapolated from select fluorophore-based data.^19,58^ Overall, our data suggest that—for a barrier-on-chip-type experiment—compound sorption is limited and not significantly impacted by the choice of the device material.

We do caution, however, that material sorption being limited (to an average of 15% even for hydrophobic compounds) naturally does not equate a complete lack of sorption. This is most clearly evidenced by fluorescence micrographs taken after completion of the experiment (including rinsing), which show red dyes (T* and F*) having diffused into the PDMS polymer matrix and being retained therein (Figure S2). With this assay method, OSTE+ and PC/PSA show clear advantages in line with prior research.^61−63^ We also quantified the release of the sorbed dye during the washes itself. At the hour timescales that are relevant for typical drug dosing regimens, this showed measurable but limited desorption (0.1–1%/h; Figure S3), again matching earlier studies.^9,11,16,17^ With both steady-state concentrations and temporal profiles thus remaining largely unaltered, we do not expect microfluidic device materials (including PDMS) to be limiting factors for pharmacokinetic and -dynamic studies on many compounds.

### 3.2 Microfluidic Tubing

As we mentioned in the Introduction, the microfluidic device is often only a small part of the entire setup. For the barrier-on-chip scenario we are modeling here, the tubing itself makes up most of the exposed fluidic area (∼7 cm^2^; the reservoirs contribute a similar-sized area but allow for use of low-sorbing plastics such as polypropylene in our case).^47^ To analyze the tubing contribution, we now consider the compound recovery from tubing-only fluidic circuits (which served as controls in Section 3.1) relative to samples exposed to the same environment in a low-sorption plastic vessel. The latter, when compared to immediately frozen samples, yield information on inherent degradation from environmental factors, which is appreciably non-zero for three compounds (between 25 and 50% for Am, Cl, and Se; Figure S1).

**Figure 3:**
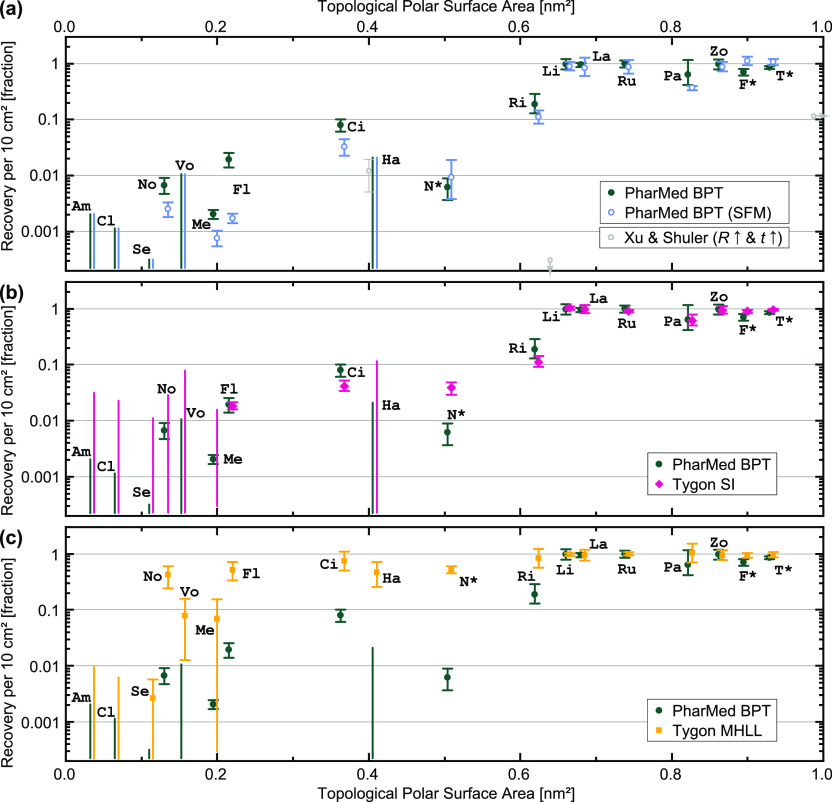
Sorption in microfluidic tubing. Compound recovery (as a fraction of thermal-degradation controls) from tubing-only flow circuits after 24 h of continuous recirculating perfusion with CCM (includes 10% serum). We compare PharMed BPT tubing with (a) same tubing, but serum-free media (SFM); (b) silicone-based tubing; and (c) MHLL-type tubing. In (a), we are additionally able to include data from one prior study, which featured higher *R* and longer exposure time *t*.^36^ All values are normalized to 10 cm^2^ tubing area to highlight the typical surface area mismatch with devices. Neuropsychopharmaca are sorted by their hydrophobicity in terms of topological polar surface area (offset for better visualization where needed); for abbreviations, see Table 1. Data are plotted as means (*n* ≥ 3 per condition), with error bars representing the 95% confidence interval. Uncapped error bars correspond to the range below the assay limit of quantification (LOQ) for those conditions where data < LOQ.

In Figure 3, we plot the compound sorption from three tubing materials: polypropylene-based (PharMed BPT; 3a), silicone-based (Tygon SI; 3b), and polyolefin-based (Tygon MHLL; 3c). We find that all three tubings show significant sorption of at least half of our study compounds, well in excess of our microfluidic devices. Some compound concentrations decrease enough to fall below our assay limit of quantitation, which is also indicated in the graphs (uncapped error bars; the affected compounds are therefore not possible to include in the earlier device analysis). We observe that sorption under all conditions depends strongly on compound hydrophobicity in terms of TPSA. Indeed, multivariate partial least squares models confirm that log(TPSA) is the most important molecular parameter across all materials (score, 1.9 ± 0.1). Out of 21 chemical properties, plus the experimental concentration parameter log(*C*/*M*), only the other hydrophobicity measures log *P* (1.7) and log *D*_7.4_ (1.5) score significantly higher than 1 on variable importance, as does the H-bond acceptor count (1.4). As examples, we plot the correlations with log *P*, log(*C*/*M*), and molar mass *M* in Figure S4 (see Analysis Section 5.5 for the full list of model parameters).

We can further quantify the sorption trend regarding TPSA using a linear regression model (in log–log space, showing the highest linearity). For the resulting data in Table 3, we normalized to a device-equivalent area of 1 cm^2^ for direct comparison with Section 3.1. These values show that not only is compound sorption higher than device sorption due to larger tubing surface areas (as represented in Figure 2*vs*Figure 3), tubing material sorption can be much higher even on an equivalent-area basis. Thus, tubing materials and the choice of tubing, are much more critical than device material choices for *in vitro* microphysiological studies of the type we mimic here. We will discuss various materials in further detail in the following paragraphs.

**Table 3:** Dependence of Compound Sorption on TPSA for Various Devices and Tubing Materials Considered in Our Study^a^

Material	slope^b^	(CI_95_)^b^	Pearson’s r
**Devices**			
PDMS	**0.08**	(0.04; 0.13)	0.76
PDMS (SFM)	**0.07**	(0.03; 0.12)	0.71
OSTE+	**0.06**	(0.00; 0.13)	0.56
PC/PSA	**0.02**	(−0.06; 0.09)	0.14
**Tubings**			
PharMed BPT	**0.25**	(0.18; 0.32)	0.89
PharMed BPT (SFM)	**0.31**	(0.21; 0.41)	0.96
Tygon SI	**0.28**	(0.19; 0.38)	0.81
Tygon MHLL	**0.10**	(−0.05; 0.25)	0.58

a For comparison purposes, all sorption values are normalized to 1 cm^2^ material surface area (*i.e.*, the device equivalent).

b Slope and the corresponding confidence interval CI_95_, in terms of log(recovery)/log(TPSA). The intercept was fixed at 100% recovery for our maximum TPSA (1 nm^2^) for all conditions.

**Figure 4:**
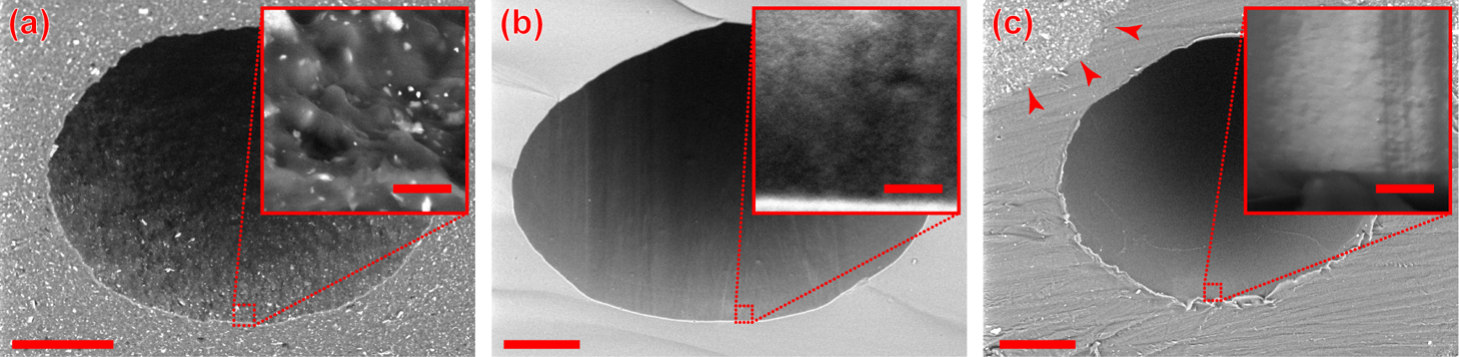
SEM images of tubing materials. Cross-section micrographs—with insets showing high-magnification views of the inner tubing surface—for (a) PharMed BPT; (b) Tygon SI; and (c) Tygon MHLL. With Tygon MHLL, the arrows indicate where the inner polyolefin (Tygon MH) core transitions into the outer PharMed BPT sheath. Scale bars are 100 μm (insets: 5 μm).

PharMed BPT (polypropylene-based) is a widely used option for *in vitro* models both by us and other groups^4,18,39,69−73^ and thus is our default choice. In Figure 3a, we first consider sorption of this material for both CCM and SFM. With SFM, five individual compounds show significantly reduced recovery (in terms of non-overlapping 95% confidence intervals), and the aggregate slope parameter for PharMed BPT increases (albeit retaining overlapping CI_95_; Table 3). With fluorophores only—interestingly, the only compounds showing increased recovery with SFM, albeit marginally—we also compare against PBS (Figure S5). This shows that PBS results track closely with SFM ones, as per our assumptions in Section 3.1 that proteins (rather than other compounds in cell culture medium) may impact sorption. The fluorophore results notwithstanding (illustrating that dyes are not necessarily ideal model compounds), and unlike with our earlier observations on PDMS, the presence of proteins does appear to passivate the surface regarding sorption for this material. In either case, PharMed BPT sorption is clearly non-negligible. This is in spite of its parent plastic (polypropylene) generally being considered low-sorption;^47^ the thermoplastic extrusion process to create a flexible material, and the plasticizers used, must thus play a role in the observed behavior. SEM on tubing cross sections (Figure 4a) provides supporting evidence for the plastic processing hypothesis. Compared to other materials, PharMed BPT features a sponge-like structure, also exhibiting a highly textured liquid-facing surface. At the very least, this increases the effective surface area of the material significantly beyond what the tubing dimensions alone imply. At worst, it may allow compound diffusion into the bulk material. Fluorescence imaging provides some support for the latter, though high autofluorescence of the material confounds the analysis (Figure S6).

We note that two prior studies have considered some aspects of PharMed BPT sorption for microfluidic applications. Xu and Shuler presented perhaps the only study focused exclusively on microfluidic tubing.^36^ Due to full parameter specification, we are able to include their data, encompassing three compounds, in Figure 3a. Their observed sorption values in CCM actually exceed ours; this would align with their lower liquid volume (thus higher *R*) and longer exposure time. Chao *et al.*, as part of an extensive *in vitro* modeling study, characterized the sorption of their entire system—including PharMed BPT (with SFM).^35^ Considering four compounds, they reported high PharMed BPT sorption (>90%) for two of them when compared to a highly specialized tubing (no longer on the market). The lack of geometric parameters (diameter, length, etc.) unfortunately does not allow for extrapolation or comparison beyond their system, but overall trends also align with ours.

Tygon SI is also utilized in the context of microphysiological models.^74−76^ Intuitively, based on the underlying plastics involved (silicone *vs* polypropylene), this should be a higher-sorbing tubing, a reasoning specifically mentioned, for example, by Bovard and Sandoz in their choice of PharMed BPT.^73^ In our study, as per Figure 3b, interestingly, only one compound (Ci) exhibits significantly lower recovery with Tygon SI, whereas one other (N*) shows a reverse behavior. The slope parameter analysis similarly shows a trend matching that of PharMed BPT. With fluorophores only, we also consider the impact of plasma pre-treatment here and find that sorption is unaffected (Figure S5; in line with our arguments in Section 3.1). In the tubing material comparison, we hypothesize that the difference in surface roughness plays a role. With Tygon SI featuring a very smooth interior surface, compared to the highly textured surface of PharMed BPT (Figure 4b), this can compensate for a presumed higher inherent silicone sorption. The latter is perhaps most clearly illustrated in the fluorescence images showing bulk absorption of red dyes (T* and N*; Figure S6). Still, based on our overall results, other practical differences should be used as the main decision factors between Tygon SI and PharMed BPT, such as the pump life (PharMed BPT), optical properties (Tygon SI), or oxygen permeability (Tygon SI).^41^

Tygon MHLL consists of an inner layer of high-purity polyolefin-based plastic (Tygon MH), coated with an outer shell of PharMed BPT to increase flexibility for peristaltic pump use (Figure 4c). We were unable to identify any *in vitro* model studies employing this tubing, potentially due to its high stiffness necessitating manual peristaltic pump adjustments when accurate flow control is desired.^41^ When compared against PharMed BPT tubing in Figure 3c, we found that Tygon MHLL performs markedly better. Recovery is significantly improved for nine compounds, and the aggregate analysis reveals a much-flattened slope (Table 3). The result is broadly in agreement with Chao *et al.*, whose aforementioned study also compared Tygon MHLL to PharMed BPT for a single compound, finding 20-fold improved recovery.^35^ Even this material, however, still suffers from compound sorption of greater than 99% for our three most hydrophobic compounds (Am, Cl, and Se; at 10 cm^2^ surface area). Lacking a better commercially available alternative, our data suggest Tygon MHLL is nonetheless the optimal peristaltic pump tubing for *in vitro* modeling with highly hydrophobic pharmaceuticals.

## 4 Conclusions

We sought to characterize sorption in microfluidic systems—uniquely focusing on *both* devices and requisite tubing—for *in vitro* studies under realistic conditions. To that end, we employed one of the largest and most hydrophobic compound panels to date, mainly consisting of neuropsychopharmaca. In contrast to many prior reports on PDMS, we did not find any compounds exhibiting 2+ fold-changes in concentration from device materials alone, attributing this in large part to discrepancies in surface-to-volume ratios. Comparing PDMS to two alternative device construction methods—OSTE+ or PC/PSA—showed that these perform at least on par if not better than PDMS. We further found that sorption of tubing materials such as the widely used PharMed BPT has a strong dependence on compound hydrophobicity, and critically dominates over device material sorption even on an area-normalized basis (let alone with typically ∼10-fold larger tubing areas). Tygon MHLL performed best among the readily available pump tubing options we evaluated.

We acknowledge that, in our combined study design, the high level of tubing sorption likely lowered our assay sensitivity for device materials alone. Thus, we hope to verify potential advantages of PC/PSA (or other microfluidic materials such as COC or glass) in future experiments with more optimized tubing circuits (see below). Regardless, our results suggest that orders-of-magnitude sorption reported with PDMS represents compound outliers more than the rule, and that prior suggested predictors are at most necessary but clearly not sufficient. To fully understand which compound properties are predictive for such “outliers,” we believe the next logical steps will lie in bringing a more theoretical perspective to bear. On the one hand, this may involve applying more advanced multivariate analysis tools that have proven themselves in for example prediction of blood–brain barrier permeability^77^ (a process that necessitates a complex combination of molecular properties, as we suspect to be the case for PDMS as well). As a starting point, a meta-analysis of existing works can suffice, but ultimately data for hundreds rather than tens of compounds may be required. On the other hand, this may involve molecular dynamics modeling to investigate the interactions of PDMS with compounds from a less reductive perspective.^78^

Overall, we see the implications of our research for biomicrofluidics—especially toward *in vitro* pharmacodynamic modeling—as three-fold: First, confirmation of compound concentrations, as well as inclusion of proper controls, is necessary. Second, all fluidic components—beyond device materials alone—need to be specified in the Materials and Methods section. Third, as Xu and Shuler already recognized,^36^ “soft” peristaltic pump-type tubing should be minimized as much as possible, replaced with glass-lined or fluoropolymer-based tubings. The advantages of peristaltic pumps are too great to fully avoid them, but the relevant tubing can be optimized (Tygon MHLL or similar) and shortened; it also provides an incentive toward micropump integration.^79^ Ultimately, tubing and associated materials clearly deserve at least the same amount of attention as device materials, if not more.

## 5 Materials and Methods

Material and equipment suppliers and item numbers are listed in Tables S1–S4.

### 5.1 Microfluidic Devices

Middle layers, identical across all device types, were prepared by cutting PC films to a suitable size with a cutting plotter and adding through-holes, as seen in Figure 1.

PDMS layers were fabricated using Sylgard 184 at 1:5 (bottom layers) or 1:20 (top layers) ratios of curing agent to base. We employed photolithographically structured molds from SU-8 on top of silicon wafers (silanized to facilitate de-molding). Bottoms were cast at ∼1 mm thickness and tops at ∼3.5 mm and cured for 60 min at 80 °C. We added inlet/outlet holes using a 2 mm diameter biopsy punch. PDMS layers were manually aligned with the PC film middle layer and clamped together in a custom-made experimental rig.

OSTE+ devices were fabricated using geometrically equivalent molds (with the above) made from aluminum by CNC milling (Teflon-coated to facilitate de-molding). Unlike the pour-over PDMS molds, OSTE+ molds were designed for injection molding after enclosure with an optically clear, non-stick film (release liner). To create inlets/outlets, and to prevent leakage, we manually added suitably shaped PDMS pieces on top of the aluminum to create full-height inlet/outlet voids and to serve as gaskets. After enclosure with the film, we clamped the mold stack with glass slides on top for added support and injected liquid OSTE+. We UV-cured the material for 180 s (bottoms; 1 mm height) or 300 s (tops; 2 mm height) at ∼10 mW/cm^–2^ and then carefully delaminated OSTE+ layers from the mold. We manually aligned them with the PD film middle layer and added gold-coated tubular rivets to the top-layer inlet/outlet holes to serve as tubing connectors. The entire stack was clamped in a custom rig and cured at 110 °C for 72+ h.

For PC/PSA devices, we first layered PSA tapes into double-height stacks to achieve a comparable 200 μm layer height. We cut channels into the layered tape using a cutting plotter and then manually aligned and bonded it to the PC film middle layer. We further bonded the tape to another PC layer to serve as the structural bottom and to a commercial microfluidic PC connector plate to serve as the structural top. A full protocol is available in Metafluidics.^80^

### 5.2 Microfluidic Setup and Tubing

All fluidic connections for device experiments utilized PharMed BPT tubing, with stainless-steel pins serving as interconnects between tubing pieces. We utilized plunger-less syringes with blunt needles as the liquid reservoirs. The liquid was guided from these into the devices and circulated through both bottom and top channels into the peristaltic pump and back into the reservoir. To ensure an equal total microfluidic flow length in each type of device, the inherently 2/3 shorter PC/tape device flows were supplemented with an additional half-channel (*i.e.*, employing a flow circuit of top–bottom–top, or bottom–top–bottom). Most tubing here featured 0.25 mm inner diameter, whereas device connections employed 0.51 mm diameter tubing more suited to our device interfaces. At 79 cm total tubing length, the average inner tubing diameter in these fluidic circuits was 0.285 mm.

Tubing-only control circuits were constructed from identical-length tubing segments (including interconnects, *etc*.), omitting only the devices themselves. For Tygon SI and Tygon MHLL, we relied solely on 0.51 and 0.38 mm inner diameter tubing, respectively, based on tubing availability. We account for the altered diameters in our analysis.

### 5.3 Test Solutions

Most test chemicals were obtained as standard solutions in suitable solvents. Where this proved infeasible, we prepared stock solutions from powders in argon-purged DMSO at the respective manufacturer-supplied solubility limits. We also used argon-purged DMSO to pre-dilute liquid standards where needed for handling. All compounds were stored according to manufacturer’s guidelines before and after use. All low-volume (<500 μL) pharmaceutical pipetting utilized low-retention tips. For intermediate handling or storage, we utilized low-binding centrifuge tubes. All other plastics used in liquid handling were either polypropylene or fluorinated ethylene propylene.

We prepared a minimal medium from MEM, supplemented with 0.2% Primocin and 10% serum replacement (omitted for SFM). PBS was prepared with 0.2% Primocin. Medium for the entire experiment was prepared as a single batch. For the assay medium, we added the test compounds at concentrations listed in Table 1 (as well as 4.5 × 10^–5^ g L^–1^ zuclopenthixol and 2.7 × 10^–4^ g L^–1^ quinine sulfate; *cf.*Analysis section), immediately prior to the experiment. The resulting DMSO and methanol concentrations were 1 and 0.33%, respectively. The medium (with or without pharmaceuticals) was allowed to equilibrate in the incubator at 37 °C under 5% CO_2_ for 30 min before being introduced to the device reservoirs.

### 5.4 Experimental Procedure

All devices and tubing (except for the no-plasma condition) were exposed to air plasma for 60 s (100 W, 1 Torr) to remove organic residues and facilitate initial wetting at −48 h (*cf.*Figure 1c). After setting up fluidics as described earlier, internal surfaces were disinfected with 70% ethanol for approximately 5 min under flow, taking care to flush out the bubbles. We subsequently rinsed the fluidics with PBS (0.2% Primocin) and continued perfusion overnight at 1% pump speed inside the incubator to calibrate the pump flow rate and check for device leakage. At −24 h, we flushed the fluidics with the medium and looped the outlet tubing into the respective inlet reservoirs for recirculating flow. Until 0 h, we primed the fluidics inside the incubator by continuous recirculation of 2 mL medium per device at a linear pump speed of 17.5 mm s^–1^. For PharMed BPT (and thus also device experiments), this corresponds to *Q* = 4.0 mL h^–1^ (Tygon MHLL: 6.0 mL h^–1^; Tygon SI: 10.8 mL h^–1^).

We intermittently (4× over 24 h) manually flushed the fluidics at high flow rates (>30 mL h^–1^) for short intervals (<10 s) to clear bubbles. At 0 h, we emptied the reservoirs and flushed the fluidics with the assay medium to minimize dilution with the blank medium. We again set up a continuous recirculating flow in the incubator, utilizing 2.5 mL assay medium per fluidic circuit at the same flow rate as previously. At 24 h (endpoint), we collected samples for analysis from each of the reservoirs, froze them immediately to −30 °C, and moved them to −75 °C for storage within hours. For transport, samples were kept on dry ice. The fluidics were subsequently rinsed with PBS at 1/10th the recirculation flow rate over 3 h.

### 5.5 Analysis

For fluorescence analysis, we relied on a plate reader. We were unable to detect quinine sulfate in any sample even after pH adjustment; it was thus omitted from further analysis. For the other compounds, we optimized λ_ex/em_ as follows (with 5/10 nm bandwidths): 501/521 (F*); 551/581 (T*); and 610/655 (N*). Calibration curves were created from serial dilutions of immediately frozen 0 h stock solutions (normalized concentration *C*’ = 1), and the corresponding “blank” medium. Based on quality-of-fit (*R*^2^ = 0.999), we chose a pseudo-sigmoidal correlation model of
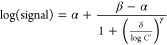
2with Greek letters denoting the fit parameters (except α, which is set at the background fluorescent signal). As with all statistical analysis (except where noted), we carried this out in OriginPro.

For LC/MS analysis, samples were analyzed at the clinical laboratory unit of the local university hospital using standard validated assays (see the next section, Clinical TDM Analysis). The zuclopenthixol assay proved highly unstable between different aliquots of the same sample and was thus excluded from further analysis.

Lastly, we cut tubing cross sections using a scalpel for SEM and fluorescence microscopy. The low-vacuum scanning electron microscope uses 15 kV acceleration voltage and records backscattered electron signals; the charge-up reduction mode allowed us to image samples without prior metallization. With the fluorescence microscope, we recorded both green- and red-channel data for tubings and devices. In all microscopy, we relied on Fiji to optimize the brightness and contrast (for fluorescence, intensities are conserved between identical materials).^81^

We initially determined the means and the corresponding 95% confidence intervals in OriginPro. We then relied on MOVER-R (implemented in Excel)^82^ to propagate these CI_95_ into the ratio-based data we present (*e.g.*, 24 h device sample over 24 h tubing sample, and so forth). For partial least square analysis, we input the molar concentration as log(*C*/*M*) as well as the following ChemAxon Chemicalize parameters:^56^ molar mass *M*, asymmetric atom count, rotatable bond count, ring count, aromatic ring count, hetero ring count, fraction of sp^3^ carbon atoms, H-bond donor count (H-bd), H-bond acceptor count, log(TPSA), polarizability, charge at pH 7.4, log *P*, log *D*_7.4_, log *S*, log *S*_7.4_, van der Waals volume, van der Waals surface area, solvent accessible surface area, minimum projection area, and maximum projection area. For molecules with multiple equilibrium states for double bond/aromatic/ring structures (*i.e.*, fluorophores), we utilize parameters from the dominant form at physiological pH. For some of these, log *S* could not be calculated, in which case we instead used the average (across analogues) as the model input.

### 5.6 Clinical TDM Analysis

All bioanalytical methods for quantification of the drugs and drug metabolites were in operation for routine analysis of human plasma and serum samples at the Therapeutic Drug Monitoring Laboratory at Karolinska University Hospital. The methods were based on LC/MS–MS, with chromatographic separation in a reversed-phase column and a triple quadrupole mass spectrometer. The methods were validated for plasma and serum samples following the EMA Guideline for bioanalytical method validation (accuracy, ±15%; precision, < 15% CV).

Sample preparation for all analytes was based on protein precipitation with methanol containing isotopically labeled internal standards for all analytes, followed by centrifugation. For Am, No, Cl, Ci, Fl, Vo, and Me, the supernatant was directly injected into the chromatographic system. For Ha, Pa, Ri, Se, and zuclopenthixol, the supernatant was evaporated under nitrogen and redissolved in 0.1% aqueous formic acid before injection. For La, Li, Ru, and Zo, the supernatant was diluted with 0.1% aqueous formic acid before injection. The chromatographic system was based on 0.1% aqueous formic acid as mobile phase A and MeOH as mobile phase B. Analytes and internal standards were monitored by one diagnostic transition in the selected reaction monitoring (SRM) mode. Calibration curves were constructed from multiple calibrators by linear regression, and acceptance criteria of sample analysis were based on internal quality control results at two levels. The LOQ was 1 nM for Ha, Pa, Ri, Se, and zuclopenthixol; 0.5 μM for La, Li, Ru, and Zo; and 5 nM for Am, No, Cl, Ci, Fl, Vo, and Me.

Low and consistent matrix effects (<15% CV), high process efficiency (>75% and above 95% for most analytes and levels), and the use of isotopically labeled standards rendered the methods acceptable for the estimation of the concentration for the analytes in the non-validated matrix used in the present drug sorption experiments.

### 5.7 PDMS Sorption Model

The model we apply for PDMS sorption was developed by Shirure and George.^58^ For a given compound *i*, it can be written as
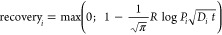
3with *R* being the ratio of microfluidic area to total volume, *P*_*i*_ the octanol–water partition coefficient (as a stand-in for the PDMS–water partition coefficient), *t* the duration of the experiment, and *D*_*i*_ the molecular diffusivity inside PDMS. The max function accounts for the lack of saturation effects in this model, an acknowledged shortcoming. Since *D*_*i*_ is even less accessible than the correct partition coefficient, the authors suggest estimating using^83^
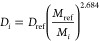
4where *M* is the molecular weight and ref refers to a reference molecule for which *D* has been measured. Shirure and George measured *D* for three fluorophores; Grant *et al.* (also in pursuit of a model) provided a recent measurement for another.^19^ It is worth noting that standard deviations of measurements are in many cases of similar magnitudes as the reported values and that eq 4 does not hold well between reference compounds. To derive the model means and error bars in Figure 3a, we assume the geometric mean between reference compounds, as well as the lowest and highest reference compound means from the literature, respectively.

## Supporting Information Available

Thermal/environmental degradation and loss of compounds; wide-field fluorescence images of microfluidic device structures and tubings; release of fluorophores from tubings and devices during post-experiment rinsing; tubing sorption as a function of other compound properties; fluorophore sorption in tubing materials; and materials and equipment (PDF)

## Abbreviations

PDMS: polydimethylsiloxane
OSTE+: off-stoichiometry thiol–ene–epoxy
PC: polycarbonate
PSA: pressure-sensitive adhesive
PET: polyethylene terephthalate
COC: cyclic olefin copolymer
CCM: complete cell culture medium
SFM: supplement-free medium
PBS: phosphate buffered saline
TDM: therapeutic drug monitoring
CI: confidence interval
SD: standard deviation
LOQ: limit of quantitation
TPSA: topological polar surface area
*P*: octanol–water partition coefficient
*R*: surface area-to-liquid volume ratio
H-Bd: H-bond donor count
TAMRA: carboxytetramethylrhodamine
SEM: scanning electron microscopy.
