# Supplementary material for: Sorption of Neuropsychopharmaca in Microfluidic Materials for In Vitro Studies
Source: ACS Appl Mater Interfaces. 2021 Sep 16;13(38):45161–74. doi: 10.1021/acsami.1c07639 (PMC8485331; doi:10.1021/acsami.1c07639)
Supplement: Supplementary file 1 — am1c07639_si_001.pdf [file am1c07639_si_001.pdf]

Supporting Information: Sorption of neuropsychopharmaca in microfluidic materials for *in-vitro* studies

Thomas E. Winkler[1,2] and Anna Herland[1,3]*

[1] Division of Micro- and Nanosystems, KTH Royal Institute of Technology, 10044 Stockholm, Sweden.

[2] Present address: Institute of Microtechnology & Center of Pharmaceutical Engineering, Technische Universität Braunschweig, 38106 Braunschweig, Germany.

[3] AIMES, Center for Integrated Medical and Engineering Science, Department of Neuroscience, Karolinska Institute, Department of Neuroscience, Karolinska Institute, 17165 Solna, Sweden.

* Email: thomas.winkler@tu-braunschweig.de

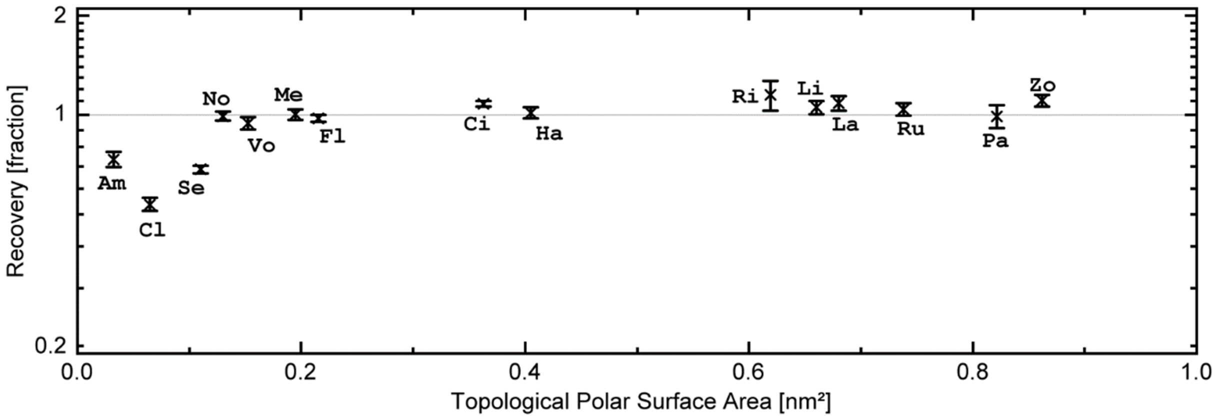

Figure S1. Thermal/environmental degradation and loss of compounds. Drug recovery from assay media aliquots stored inside the incubator alongside the 24 h device experiments, normalized to immediately-frozen controls. All compounds are sorted by their hydrophobicity in terms of topological polar surface area; for abbreviations see Table 1. Data are plotted as means ± standard deviation (initial: n=4; 24 h: n=8). The three most hydrophobic compounds (Am, Cl, Se) exhibit significant inherent losses.

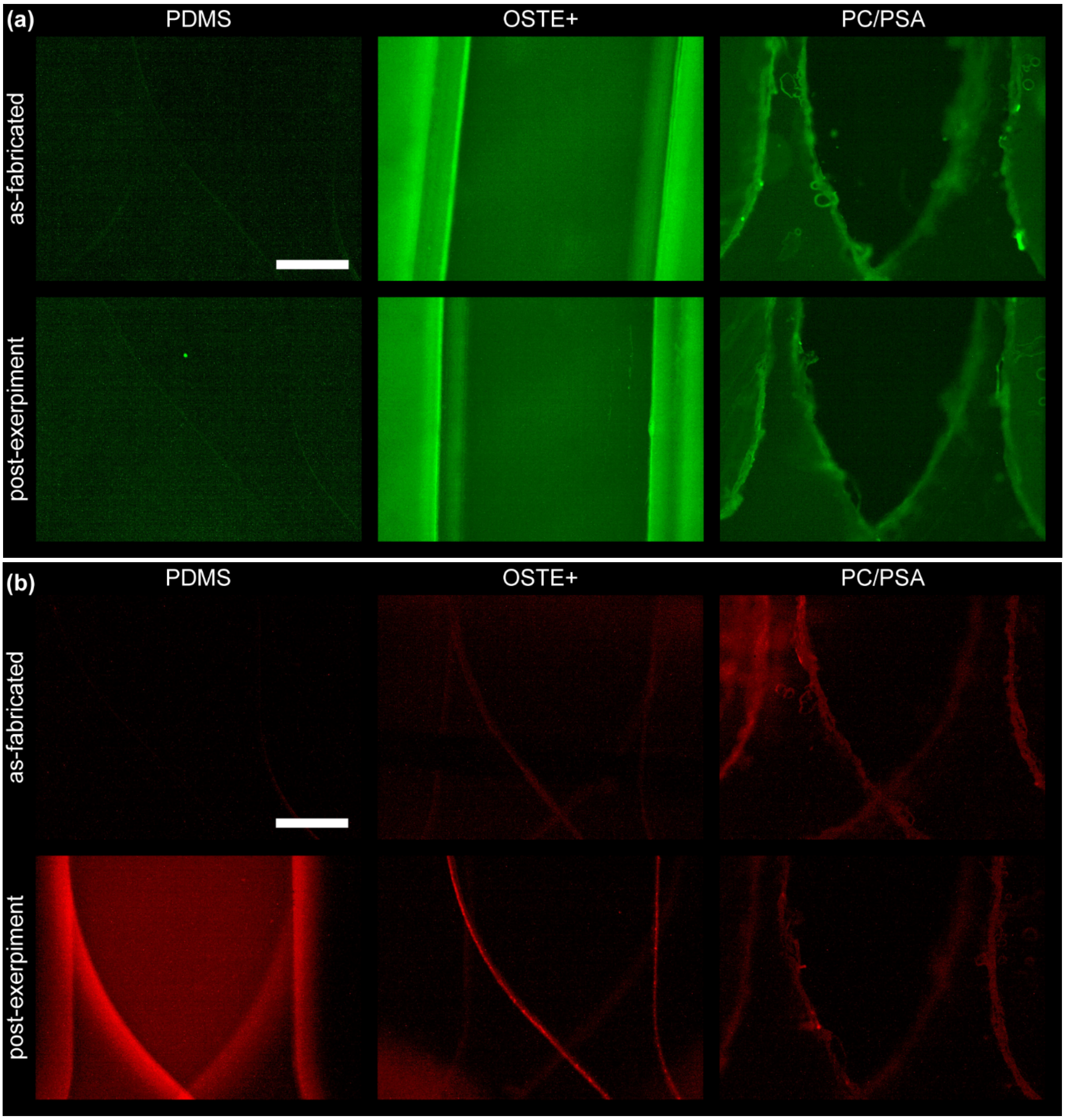

Figure S2. Widefield fluorescent images in green (**a**; F*) and red (**b**; T*, N*) of the microfluidic device structures from the various materials (columns), either as-fabricated or post-experiment (rows). PDMS shows a very weak increase in green fluorescence post-experiment; red fluorescence conversely increases markedly, with clear diffusion of the dye into the polymer matrix. OSTE+ also shows an increase in red fluorescence, but of lower magnitude and only at the surfaces (no diffusion into the bulk), in agreement with prior studies.[61–63] Material autofluorescence in the as-fabricated state eliminates the possibility of picture-based sorption analysis for OSTE+ (green). The same applies to PC/PSA (green, red), though sorption-based red fluorescence is clearly less than for PDMS. Note that autofluorescence inside the channel (where cells would be located in Organ-on-Chip experiments) is low enough to still allow for high-quality imaging (particularly confocal) as demonstrated by us and others.[39,46] Scale bars: 500 μm.

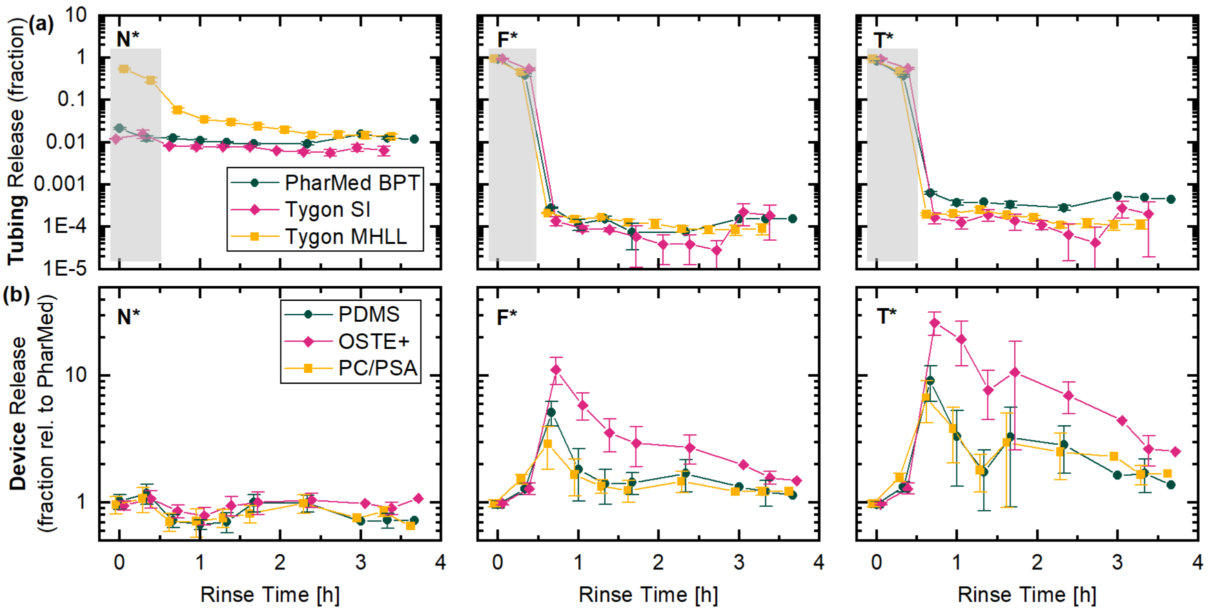

Figure S3. Release of fluorophores from **(a)** tubing materials and **(b)** devices during post-experiment rinsing with PBS. The 0 h data points correspond to those shown in Figures 2 and 3. Unlike those Figures, however, data here are plotted as means ± standard deviation (devices: n=4; tubings: n=3). In the first 30 minutes (gray shaded area), remaining dye solution is flushed out of the system. Subsequent data illustrate release of sorbed compounds. With tubings, N* is the only one with appreciable amounts. With devices, data are normalized to PharMed BPT tubing, *i.e.*, a 10-fold increase for T* corresponds to 10×0.0005 = 0.5% concentration fraction. N* release is below the detection limit here (in part due to the high tubing contribution), but F* and T* do show measurable compound release. We note that the high OSTE+ release is likely an artifact from how tubing-to-device interfaces are constructed. These devices (unlike both PDMS and PC/PSA construction) feature recessed spaces between the tubing and the OSTE+, spaces that would not experience significant convective flow. Thus, dye solution in those spaces would only comparatively slowly diffuse into the convective flow path, compared to the other material devices.

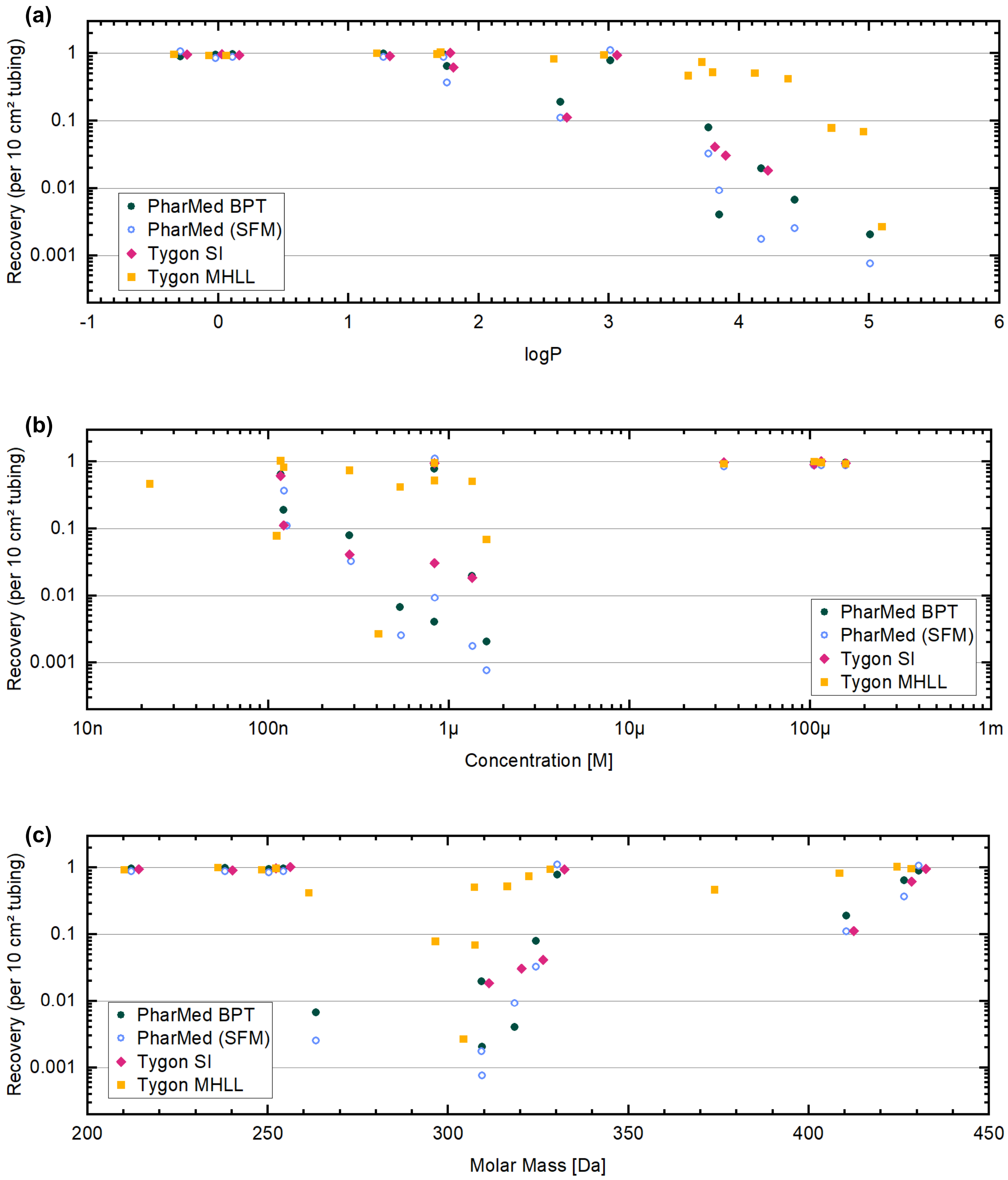

Figure S4. Tubing sorption as a function of compound properties. We re-plot data from Figure 3b-c as a function not of TPSA but of **(a)** log*P*, **(b)** molar concentration *C/M*, and *(c)* molar mass *M*. For clarity, we plot mean values only. Correlation of recovery with log*P* is almost as clear as with log(TPSA) in Figure 2, while other factors such as concentration or mass do not show clear correlations.

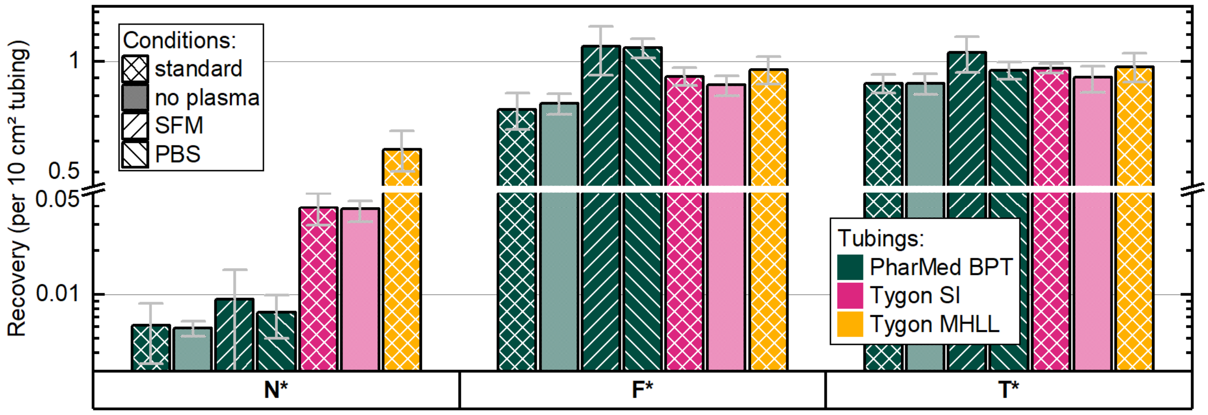

Figure S5. Fluorophore sorption in tubing materials. The various materials (colors) are assessed under standard conditions, as well as certain adaptations: omission of plasma pre-treatment (no plasma), or replacement of CCM with either SFM or PBS. Control and SFM data correspond to that shown in Figure 3. Data are plotted as means ($n \geq 3$ per condition), with error bars representing the 95% confidence interval. Note the break in the y-axis for better visualization of within-group differences. Inclusion or omission of plasma treatment does not show any impact on sorption. For fluorophores, lack of proteins appears to lead to increased recovery; the differences within protein-free solutions (SFM and PBS) are smaller.

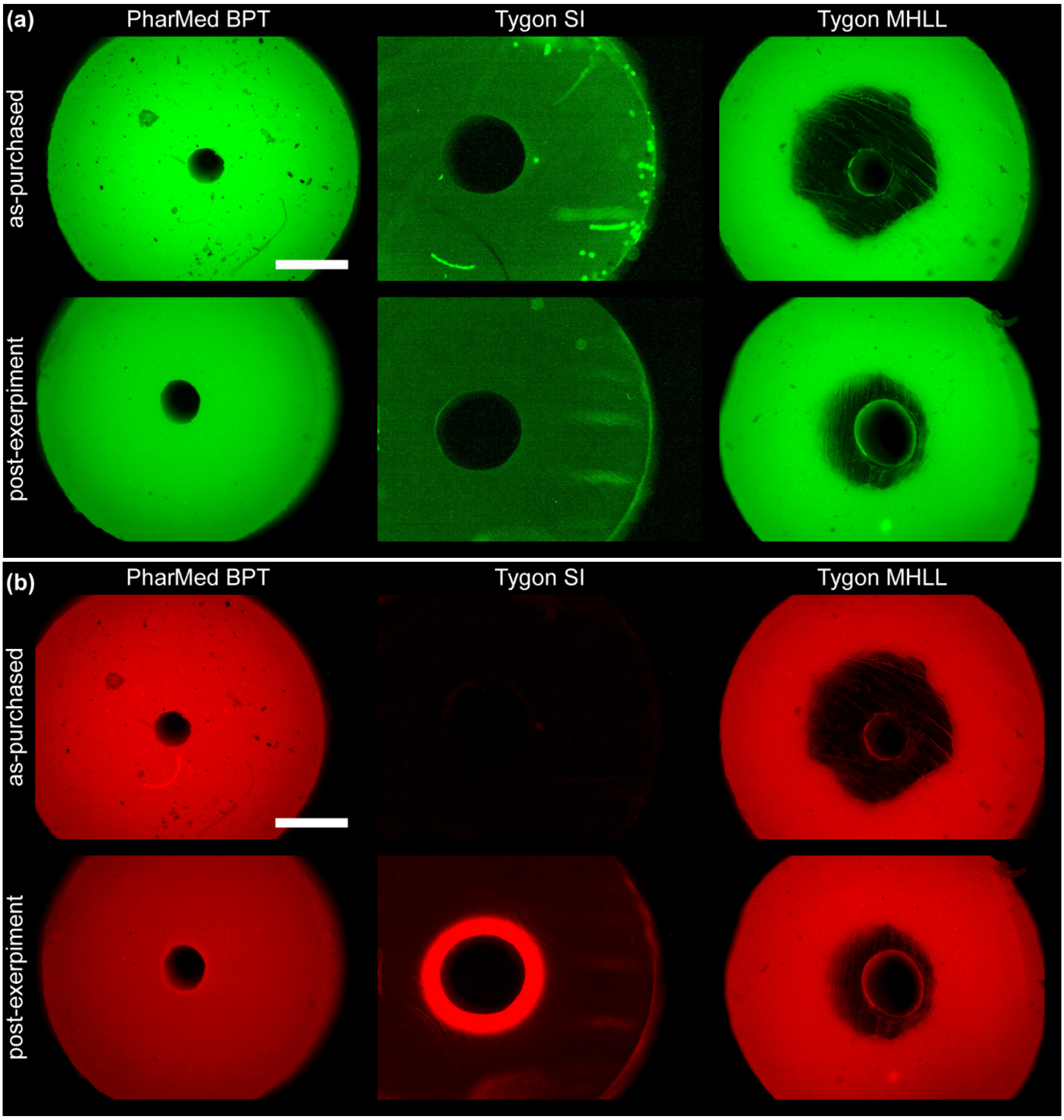

Figure S6. Widefield fluorescent images in green (**a**; F*) and red (**b**; T*, N*) of the tubings from the various materials (columns), either as-purchased or post-experiment (rows). Black or highly fluorescent specks are artifacts from cross-sectioning. The clearest indication of material sorption is with Tygon SI showing diffusion of red dye into the polymer matrix (in line with PDMS device results). The pictures suggest a small increase in surface adsorption (evidenced by a thin, sharply delineated layer) for Tygon MHLL (green & red), as well as potentially for Tygon SI (green). PharMed BPT may even exhibit some bulk absorption (red; evidenced by a more diffuse ring of fluorescence). However, material autofluorescence in the as-purchased state eliminates the possibility of reliable picture-based sorption analysis for PharMed BPT (green & red) and Tygon SI (green). The PharMed BPT sheath of Tygon MHLL tubing is naturally also autofluorescent. Scale bars: 500 µm.

Materials & Equipment

Table S1. Neuropsychopharmaca & Fluorophores

Product	Manufacturer	Identifier
Amitriptyline (Am) 1 mg/ml in Methanol	Cerilliant	A-923
Citalopram (Ci) 0.1 mg/ml in Methanol	Cerilliant	C-057
Clomipramine (Cl) 1 mg/ml in Methanol	Cerilliant	C-118
Fluoxetine (Fl) 1 mg/ml in Methanol	Cerilliant	F-918
Haloperidol (Ha) 1 mg/ml in Methanol	Cerilliant	H-030
Lacosamide (La) powder	European Pharmacopoeia (EP)	Y0001982
Licarbazepine (Li) powder	Cayman Chemical	18467
Methadone (Me) 1 mg/ml in Methanol	Cerilliant	M-007
Nortriptyline (No) 1 mg/ml in Methanol	Cerilliant	N-907
Paliperidone (Pa) 1 mg/ml in Methanol	Cerilliant	H-076
Risperidone (Ri) 1 mg/ml in Methanol	Cerilliant	R-006
Rufinamide (Ru) powder	Cayman Chemical	18870
Sertraline (Se) 1 mg/ml in Methanol	Cerilliant	S-021
Vortioxetine (Vo) powder	Cayman Chemical	30183
Zonisamide (Zo) powder	Cayman Chemical	24183
Zuclopenthixol powder	Cayman Chemical	24961
Reference Dye Sampler Kit (1mM; includes quinine sulfate, fluorescein, carboxy-TRITC, sulforhodamine 101, nile blue)	Invitrogen	R14782
Certified TDM radio-labeled reference samples	Merck and Toronto Research Chemicals	(N/A)

Table S2. Other Reagents

Product	Manufacturer	Identifier
Dimethyl sulfoxide	Sigma Aldrich	D2650
DPBS, − calcium, − magnesium	Gibco	14190144
Minimum Essential Medium (MEM), no glutamine, no phenol red	Gibco	51200046
KnockOut Serum Replacement	Gibco	10828010
Primocin	Invivogen	ant-pm

Table S3. Microfluidics

Product	Manufacturer	Identifier
PDMS Silicone Elastomer Kit	DOW	Sylgard 184
OSTE+ Crystal Clear	Mercene Labs	OSTEMER 322
OSTE+ Release Liner	3M	9742
Tubular rivets, gold plated (DIN 7340 A 2.5×0.3×3.5 mm)	Kaiser Waltermann	(N/A)
Double Sided Medical Tape	3M	9877
Microfluidic interface (polycarbonate, microscopy slide format, 2x16 olives)	Microfluidic chipshop	10-1121-0343-03
Polycarbonate film (125 µm)	Covestro	Makrofol DE 1-1

Syringes, 5ml (as liquid reservoirs)	Restek	22774
Blunt dispensing needles 23G	Metcal	923050-TE
Steel Microfluidic Fittings 23G	Elveflow	LVF-KFI-13
PharMed BPT peristaltic pump tubing	Ismatec	SC0320
PharMed extension tubing	Ismatec	SC0337
PharMed extension tubing	Ismatec	SC0339
Tygon MHLL peristaltic pump tubing	Ismatec	SC0716
Tygon SI peristaltic pump tubing	Ismatec	SC0620

Table S4. Equipment

**Product**	**Manufacturer**	**Identifier**
Low-pressure plasma system	Diener	Femto
Cutting Plotter	Graphtec	CE5000
16-channel peristaltic pump with click-‘n-go cartridges	Ismatec	IPC-N
CO_2 incubator	Thermo Scientific	Heracell Vios 160i
Multimode Plate Reader	Tecan	Infinite M1000 Pro
Tabletop SEM	Hitachi	TM-1000
Fluorescent microscope with GFP and Texas Red filters	Zeiss	Axio Scope.A1
Dionex Ultimate 3000	Thermo Scientific	Dionex 3000
Quantiva or Quantis Triple-Stage Quadrupole Mass Spectrometer	Thermo Scientific	Quantiva or Quantis
Hypersil Gold C18 reversed phase column (50×2.1 mm, 1,9µm)	Thermo Scientific	25002-052130
